# Non-Anticoagulant Heparins Are Hepcidin Antagonists for the Treatment of Anemia

**DOI:** 10.3390/molecules22040598

**Published:** 2017-04-08

**Authors:** Maura Poli, Michela Asperti, Paola Ruzzenenti, Annamaria Naggi, Paolo Arosio

**Affiliations:** 1 Department of Molecular and Translational Medicine, University of Brescia, Viale Europa 11, 25123 Brescia, Italy; maura.poli@unibs.it (M.P.); michela.asperti@unibs.it (M.A.); p.ruzzenenti001@unibs.it (P.R.); 2 G. Ronzoni Institute for Chemical and Biochemical Research, Milan 20133, Italy; naggi@ronzoni.it

**Keywords:** heparin, hepcidin, iron homeostasis, anemia

## Abstract

The peptide hormone hepcidin is a key controller of systemic iron homeostasis, and its expression in the liver is mainly regulated by bone morphogenetic proteins (BMPs), which are heparin binding proteins. In fact, heparins are strong suppressors of hepcidin expression in hepatic cell lines that act by inhibiting the phosphorylation of SMAD1/5/8 proteins elicited by the BMPs. The inhibitory effect of heparins has been demonstrated in cells and in mice, where subcutaneous injections of non-anticoagulant heparins inhibited liver hepcidin expression and increased iron bioavailability. The chemical characteristics for high anti-hepcidin activity in vitro and in vivo include the 2O-and 6O-sulfation and a molecular weight above 7 kDa. The most potent heparins have been found to be the super-sulfated ones, active in hepcidin suppression with a molecular weight as low as 4 kDa. Moreover, the alteration of endogenous heparan sulfates has been found to cause a reduction in hepcidin expression in vitro and in vivo, indicating that heparins act by interfering with the interaction between BMPs and components of the complex involved in the activation of the BMP/SMAD1/5/8 pathway. This review summarizes recent findings on the anti-hepcidin activity of heparins and their possible use for the treatment of anemia caused by hepcidin excess, including the anemia of chronic diseases.

## 1. Introduction

The biological function of heparins has not been fully established yet, but it is well known that they can bind a large number of plasma proteins with important biological roles that include growth factors, morphogens, and cytokines. This occurs because heparin shares the same binding capacity as the heparan sulfates (HSs) bound to the surfaces of all mammalian cells. The binding of growth factors and morphogens to surface HSs is important to modulate and control their functionalities, availability, and stability [1]. Most members of the TGF-beta superfamily bind heparin and HSs, and they include more than 15 types of bone morphogenetic proteins (BMPs) [2]. Among them, BMP2 and BMP4 and the homologous drosophila decapentaplegic have been extensively characterized for the binding to heparin and to the endogenous heparan sulfates, an interaction shown to be essential both for making a gradient during embryo development and for controlling local concentration [3]. The heparan sulfates have a major role for the binding and activity of FGF and VEGF and for the osteogenic activity of BMPs [2,4,5]. More recently, it was shown that the BMPs, and in particular BMP6, in the liver have the specific role of activating the expression of hepcidin, the iron-inflammation peptide hormone that regulates systemic iron homeostasis [6]. This has stimulated studies to verify if heparin can interfere with the activity of BMP6 and hepcidin expression in cells and in animals, and this led to the demonstration that non-anticoagulant heparins are efficient suppressors of hepcidin. This review summarizes the recent development on mammalian iron homeostasis, its regulation and pathological deregulations, and the possible use of heparins for treatment of anemias caused by hepcidin excess, as it occurs in inflammatory conditions. 

## 2. Iron Homeostasis and the Role of Hepcidin

Iron is an essential micronutrient for all organisms since it acts as a cofactor for enzymes involved in vital processes including oxygen transport (hemoglobin and myoglobin), citric acid cycle and cellular respiration (Fe/S cluster proteins and cytochromes), antioxidant defense (peroxidase and catalase), DNA/RNA synthesis, and nucleotide metabolism (ribosome reductase). However, it is also potentially toxic because Fe(II) can participate in Fenton’s reaction, giving rise to toxic oxygen species. As a consequence, iron homeostasis must be tightly controlled, at both the cellular and the systemic levels. The mechanism acting at the cellular level has been clarified long ago and uses the iron regulatory proteins that bind elements on the ferritin and transferrin-receptor-1 mRNA in an iron-dependent manner and that thus regulate iron storage and iron uptake in the opposite way [7]. The study of systemic iron homeostasis was more complex, and the basic mechanism has only recently been elucidated. The normal Western daily diet contains about 10–15 mg of iron, most of which is heme iron and the rest is as Fe(III) complexed to various molecules, but only a portion of this iron is absorbed to compensate the physiological losses of the body (1–2 mg/day). They are not regulated and consist in cell defoliation, sweat, and by periodic/occasional blood losses that must be balanced by an equal amount of iron intake to maintain the 4–5 g of iron needed for the synthesis of hemoglobin and the many essential iron enzymes [8]. Only under conditions of iron deprivation, most of the available iron can be taken up by the body. The mechanism used by heme iron to enter the duodenal enterocyte has not been clarified, while non-heme iron is first reduced by an epithelial ferric reductase DcytB that makes it more soluble and adapt to be taken up by the transporter named DMT1 [9]. Once in the enterocyte, the iron can enter the storage compartment of the ferritin to be lost at the end of the cell life cycle, or be transferred to circulation via the exporter named ferroportin, in a step that needs the assistance of a ferroxidase enzyme, hephestin, or ceruloplasmin to load it onto the serum transferrin. The transferrin iron is delivered via the transferrin-receptor-1 to the various organs and in particular to the bone marrow where the erythroid precursors use most of it for hemoglobin synthesis [10]. The hemoglobin iron is eventually released to the transferrin, and the red blood cells are thereafter taken up by phagocytic macrophages, mainly in the spleen, where the heme is degraded by heme-oxygenase and the iron is exported via the ferroportin. This pathway implies that the availability of systemic iron relies mostly on the cellular iron export that depends on the ferroportin, and the exported iron originates partly from the diet (1–2 mg/die) and mostly from hemoglobin recycling (20–25 mg/die). In fact, the major control of systemic iron relies on a protein hormone, named hepcidin, that binds specifically to ferroportin to induce its internalization, ubiquitination, and degradation, thus reducing systemic iron availability [11]. When hepcidin is low, iron is readily released by enterocytes and macrophages, it becomes more available and may determine iron overload in the parenchymal cells, as it occurs in hereditary hemochromatosis. If hepcidin is high, less iron is absorbed and recycled, leading to anemia and iron retention in the macrophages of the spleen. After the discovery that hepcidin is the major controller of systemic iron availability, the focus was on the mechanism of its regulation. Hepcidin is produced as a propeptide that is processed in the mature 25 amino acid form stabilized by four disulfide bonds. It is expressed mainly by the hepatocytes in an iron-dependent manner with a typical feedback manner: upregulated when body iron is high and downregulated when body iron is low. The finding that hepcidin is upregulated also by inflammation and downregulated by erythroid activity and hypoxia was important [12]. The important role of inflammation on hepcidin transcription clarified why many inflammatory conditions are accompanied by low hemoglobin, as it occurs in the anemia of chronic diseases or anemia of inflammation [13]. Dysregulations of hepcidin expression are associated with various disorders, so an understanding of the detailed mechanism of hepcidin control could lead to the development of therapies for the treatment of various iron-related disorders.

The regulation of hepcidin occurs mainly at a transcriptional level and mostly relies on the BMP6/SMAD pathway that, when activated, strongly stimulates hepcidin expression in the liver [14]. This involves the BMP receptors and requires the assistance of a specific co-receptor named hemojuvelin (HJV) that is a GPI-anchor membrane protein [15]. The BMP binding activates the Type II receptor BMPRII to phosphorylate the Type I receptor ALK2, which causes the phosphorylation of SMAD1/5/8 that then associates with SMAD4 and the complex migrates to the nucleus to bind the element at the hepcidin promoter [16]. Iron activates this pathway via a mechanism that has not been fully elucidated but is known to involve the induction of BMP6 [17]. The inflammatory stimulus acts mainly via IL6 produced by macrophages that activates the JAK/STAT3 pathway, which potentiates the BMP/SMAD pathway [18]. A further regulation is made by the presence of a liver-specific membrane serine-protease named TMPRSS6, which cleaves and inactivates HJV, thus inhibiting hepcidin expression [19]. Mutation of this gene cause a genetic iron-refractory iron-deficiency anemia (IRIDA) because of elevated hepcidin levels [20]. 

## 3. Heparins and Hepcidin Expression

In vitro studies on hepatic cells initially showed that BMP2 is a strong inducer of hepcidin, but later it was found that BMP6 is the physiological BMP dedicated to hepcidin expression based on the evidence that the major phenotype of mice deficient in BMP6 was a massive liver iron overload and that BMP6 is regulated in an iron-dependent manner [21]. After these findings, many approaches have been taken for a pharmacological control of hepcidin expression, identifying and developing both hepcidin agonists and antagonists. These studies have been described in recent reviews and include molecules that sequester hepcidin, that interfere with the BMP/SMAD or IL6/STAT3 pathways, that regulate hepcidin expression, that act on BMP co-receptors, and that mimic hepcidin and others [22,23,24]. 

Our approach started with the observation that BMPs are heparin binding proteins and thus heparin might interfere with the BMP/SMAD pathway that controls hepcidin expression. We demonstrated that commercial heparins used in clinics for their anticoagulant property are strong inhibitors of hepcidin expression in vitro in mice and in the few hospitalized patients we analyzed [25]. In vitro, we tested hepatoma HepG2 cells with unfractionated heparin (UFH), low-molecular-weight heparin (LMWH), and the pentasaccharide Fondaparinux, and we observed that UFH was the most effective in suppressing hepcidin expression at pharmacological concentrations with an effect that lasted up to 22 h. Moreover, in these cells, UFH fully suppressed hepcidin stimulation by exogenous BMP6 in a manner slightly different from that of BMP2. LMWH maintained some anti-hepcidin activity but at higher doses, while the pentasaccharide Fondaparinux was only marginally functional; thus, the potency was in the following order: UFH > LMWH >> Fondaparinux [25]. Mice treatments with pharmacological concentrations of UFH downregulated hepcidin and modified body iron status, with an increase of circulating iron and a decrease of spleen storage iron [25]. However, the use of heparin in mice was difficult because of its anticoagulant activity. To overcome this problem, the heparins were chemically modified to reduce/abolish this activity by altering the antithrombin binding site. This was done by a process of oxidation and reduction that produced Glycol-split heparins or by increasing the sulfation degree in the super-sulfated heparins [26]. The Glycol-split heparins we analyzed, named RO-82 and RO-68, were completely devoid of anti-coagulant activity and could be easily used in the mice without side effects, showing a potent hepcidin inhibitory activity [27]. The super-sulfated heparin, coded SSLMWH-19, was even more potent than the glycol-split heparins although it retained a marginal anticoagulant activity [28]. The anti-hepcidin activity of heparins, both anti-coagulant and modified ones, was always accompanied by the concomitant reduction in the pSMAD activation and the reduction in Id1 mRNA, a marker of this pathway, both in hepatoma cells and in mice. In addition, we observed that the inhibitory effect was specific for the BMP/SMAD pathway; in fact, when we stimulated hepcidin expression with an inflammatory stimulus (IL6 in cells and lipopolysaccharide in mice), heparins suppressed hepcidin induction by inhibiting only the BMP-SMAD related pathway, without any changes in the activation of the pSTAT3 inflammatory pathway [27].

**Figure 1:**
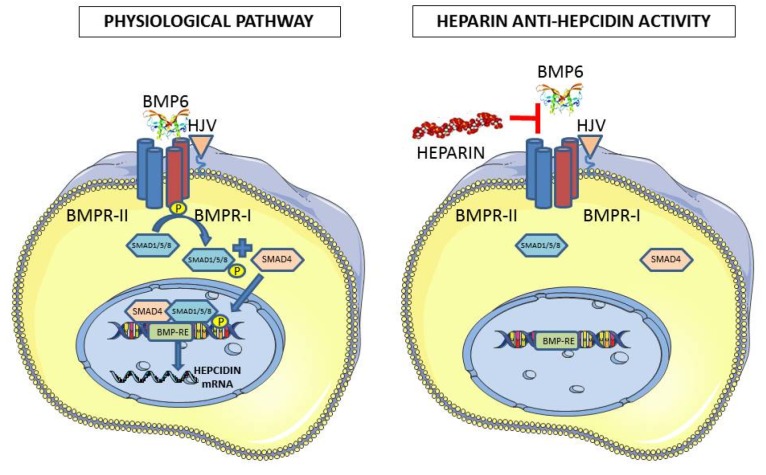
Scheme of the anti-hepcidin activity of heparin. In the physiological pathway, the binding of BMP6 causes the phosphorylation of Type I receptor by Type II receptor, which phosphorylates SMAD1/5/8 that associates with SMAD4, and the complex enters the nucleus to bind to the responsive element on the hepcidin promoter. The anti-hepcidin activity of heparin is thought to act by sequestering BMP6 and interfering with its binding to the receptors.

Molecular weight and the degree of sulfation are the two major chemical properties of heparins that we analyzed to evaluate their effect on anti-hepcidin activity. Heparin preparations are a pool of molecules with different molecular weights. Thus, they were fractionated on gel filtration columns to obtain preparations with defined and restricted molecular weight. We found that the Glycol-split heparins with a molecular weight above 7 kDa were able to completely suppress hepcidin expression in hepatoma cells, even after BMP6 stimulation. Interestingly, the super-sulfated heparin SSLMWH-19 preserved a high anti-hepcidin activity even with molecular weight as low as 4 kDa. In vivo experiments in mice confirmed that the Glycol-split heparins above 7 kDa and the super-sulfated ones of about 4 kDa were highly effective in suppressing liver hepcidin expression [29]. We further analyzed heparins in which the sulfated groups in position 6-O and 2-O were selectively removed or were N-acetylated and we found that they had a reduced inhibitory effect on hepcidin expression both in the absence or presence of BMP6 stimulation and in mice [29]. Altogether, we showed that non-anti-coagulant Glycol-split heparins have a strong anti-hepcidin property, and to be functional they must be 2O- and 6O-sulfated and have a molecular weight > 7 kDa that corresponds to about 17 saccharide residues. Similarly, the effective super-sulfated heparins have a molecular weight >4 kDa and are made of more than 9 saccharide residues. This is in line with the finding that to exert a potent activity, these compounds should expose numerous binding sites for the interaction with different molecules or different sites of the same molecule. The heparins used in our studies and their properties are summarized in Table 1, and a scheme of the mechanism of the anti-hepcidin activity of heparin shown in Figure 1.

## 4. Alternative Ways of Heparin Administration

Heparin is normally administrated subcutaneously, the absorption of which is now well characterized and known [30]. Therefore, for the treatment of mice, we use the same subcutaneous administration that produced good results in the short run [27]. However, in some cases of iron deficient anemia caused by hepcidin excess, it would take a long time to replenish the iron stores, and thus chronic treatments should probably be used. Thus, alternative ways of administration should be explored—methods that are less invasive than the daily subcutaneous injection. To this aim, we started using mice osmotic pumps, which ensure the continuous delivery of the compound for 7 or 28 days. This has already been successfully used in mice for the delivery of a non-anticoagulant heparin to study its anti-tumor activity [31,32]. This encouraged us to deliver Glycol-split heparins with an osmotic pump in mice for 7 days. The preliminary data showed that the implants and heparin delivery did not cause any evident adverse effect, but we did find a significant inhibition of liver hepcidin mRNA and of the serum hepcidin (unpublished data). These results encourage use to continue with further analysis. Oral administration of heparin is also interesting; oral heparin therapeutics for anticoagulant activity using different methods for drug delivery, such as liposomes, emulsions, or chemically modified heparin, has long been attempted, without major effects [33]. In preliminary attempts, we administrated the super-sulfated SSLMWH-19 heparin by a single oral gavage in mice and observed a significant reduction in liver hepcidin mRNA. Further studies are needed to verify if functional, low-molecular-weight heparin can diffuse across the gastrointestinal membrane and enter the circulation to exert reproducible anti-hepcidin activity.

## 5. Hepcidin and Endogenous Heparan Sulfates

**Figure 2:**
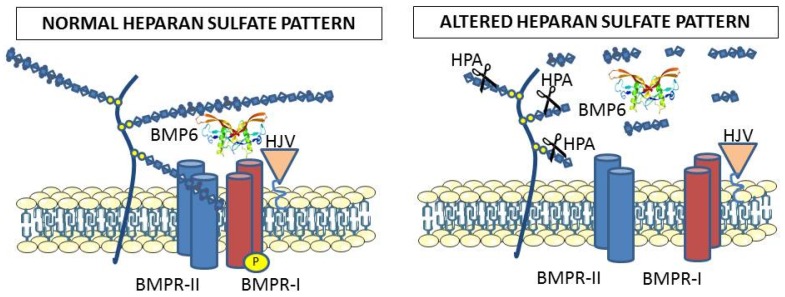
The heparan sulfates bound to proteoglycans cover cellular membranes and are thought to participate in the binding of BMP6 to the receptors for activation of the SMAD signaling. This is supported by the evidence that alteration of the endogenous heparan sulfate structure by heparanase overexpression strongly reduced BMP6 signaling and hepcidin expression in cells and mice. Exogenous heparins probably interfere with the roles of endogenous heparan sulfates.

The exogenous heparin is normally used to interfere with the physiological interactions between the ligands and endogenous heparan sulfates, thus it was of interest to verify if and how the heparan sulfates participate in the mechanism of hepcidin expression. As an approach to study this, we analyzed the effect of heparanase, the enzyme that physiologically degrades the heparan sulfates [34]. Overexpression of the enzyme in hepatic cell lines caused an inhibition of hepcidin expression and an increase of cellular iron and ferritin [35]. Mice with overexpression of heparanase were healthy, but they showed abnormal levels of hepcidin and liver iron loading [35]. In another approach, we treated HepG2 cells with sodium chlorate, a known inhibitor of heparan sulfate biosynthesis that interferes with the sulfate carrier donor PAPS (3’-phosphoadenosine 5’-phosphosulfate). This caused a strong inhibition of hepcidin expression even after stimulation with BMP6 (unpublished results). Experiments to inhibit key enzymes of heparan sulfate biosynthesis in cells and in mice are in progress. We propose a scheme of the mechanism of action of endogenous heparan sulfates involvement in hepcidin expression pathway, as shown in Figure 2. 

## 6. BMP6 and Heparin Binding

Most BMPs bind heparin, and in fact they were originally purified from heparin columns [36]. A major heparin binding domain has been identified in BMP2 by site-directed mutagenesis [37,38,39], and it involves the N-terminus that is disordered and not detected in the crystallographic structure. The site does not overlap with the binding sites of the receptors that are at the edges of the molecule [40]. The sequence is conserved also in BMP4, and the biological activity of both molecules is affected by exogenous heparins, although the effect seems to be variable stimulatory [41] or inhibitory [42]. This N-terminal sequence is substituted in BMP6 and BMP7, being longer and with a lower density of basic residues. More important in our study is that this segment is absent in the commercial preparations of BMP6 that are biologically active, possibly because the basic residues interfere with expression and purification. Our preliminary data indicate that this sequence is important for heparin binding, and suggest the presence of a second heparin binding site exposed on the opposite site of the molecule that has lower affinity. Moreover, the BMP receptors were found to bind heparin [42], and it was proposed that they may bind Type II receptor and facilitate its interaction with Type I [3], thus acting as a co-receptors, similar to what has been described for Neogenin, which also binds to Type II before Type I [40]. The binding of HJV to heparin has not been studied yet. 

## 7. Conclusions

Heparin interferes with the BMP6/SMAD pathway of hepcidin regulation, acting as a strong suppressor. The activity is also evident in vivo in animal models, and non-anticoagulant heparins are promising heparin antagonists that can be used for treatments of conditions with an excess of hepcidin, such as the anemia of chronic disease, also known as anemia of inflammation, which is the most common form of anemia in hospitalized patients, and the iron-refractory iron-deficient anemia (IRIDA) mainly linked to genetic variations of the Tmprrs6 gene. An understanding of how heparin and heparan sulfates participate in the mechanism of BMP/SMAD pathways is emerging, but awaits clarification from further studies.

## Figures and Tables

**Table 1:** List of the heparins tested for the anti-hepcidin property in vitro and/or in vivo. These include anti-coagulant and non-anticoagulant heparins as indicated. The main features and the anti-hepcidin potency (*** strong anti-hepcidin activity/** intermediate anti-hepcidin activity/* low anti-hepcidin activity) of the heparins are described. The fractions of fractionated heparins are coded as F plus a number.

	Heparins Tested for Anti-Hepcidin Activity				
Compounds	Description	Mw (kD)	Anticoagulant	Potency	Ref.
UFH	Pig Mucosal heparin, commercial (Calciparina)	12.0–15.0	yes	***	[25,27]
PMH	Pig Mucosal heparin (API)	19.9	yes	***	[25,27]
LMWH	Commercial LMWH Enoxaparin (Clexane)	4.5	yes	**	[25,27]
FONDAPARINUX	Commercial pentasaccharide (Arixtra)	1.7	yes	*	[25,27]
RO-82	Glycol-Split,	16.0	no	***	[27,29]
RO-68	Partially 2O-desulfated,Glycol-split	16.4	no	***	[27,29]
NAc-91	N-acetylated	16.0	no	*	[27,29]
NAc-RO-00	N-Acetylated, glycol-split	15.9	no	*	[27,29]
SSLMWH-19	Super-sulfated LMW	8.8	partially	***	[27,29]
PMH-F1	PMH fraction	21.6	yes	***	[29]
PMH-F2	PMH fraction	14.4	yes	***	[29]
PMH-F3	PMH fraction	10.0	yes	***	[29]
RO-82-F1	Glycol-Split, fraction	12.0	no	***	[29]
RO-82-F2	Glycol-Split, fraction	9.2	no	***	[29]
RO-82-F3	Glycol-Split, fraction	7.8	no	**	[29]
RO-82-F4	Glycol-Split, fraction	6.8	no	**	[29]
RO-68-F1	Partially 2O-desulfated Glycol-split	7.8	no	**	[29]
RO-68-F2	Partially 2O-desulfated Glycol-split	6.2	no	**	[29]
RO-68-F3	Partially 2O-desulfated Glycol-split	3.9	no	*	[29]
SSLMWH-19-F1	Super-sulfated LMW fraction	12.9	partially	***	[29]
SSLMWH-19-F2	Super-sulfated LMW fraction	10.3	partially	***	[29]
SSLMWH-19-F3	Super-sulfated LMW fraction	6.9	partially	***	[29]
SSLMWH-19-F4	Super-sulfated LMW fraction	4.0	partially	***	[29]
2-O	PMH 2-O desulfated	-	no	*	[29]
6-O	PMH 6-O desulfated	-	no	*	[29]
